# Management in tension: the Brazilian public health surveillance system and its response to the covid-19 pandemic

**DOI:** 10.1590/S0104-59702023000100040

**Published:** 2023-09-04

**Authors:** Catalina Kiss, Carlos Henrique Assunção Paiva, Luiz Antonio Teixeira

**Affiliations:** i Bolsista do Programa de Incentivo ao Desenvolvimento Institucional, Departamento de Pesquisa em História das Ciências e da Saúde/Casa de Oswaldo Cruz/Fiocruz.Rio de Janeiro – RJ – Brasil catalinakiss9@gmail.com; ii Coordenador do Observatório História e Saúde, Departamento de Pesquisa em História das Ciências e da Saúde/Casa de Oswaldo Cruz/Fiocruz.Rio de Janeiro – RJ – Brasil carlos.paiva@fiocruz.br; iii Pesquisador, Departamento de Pesquisa em História das Ciências e da Saúde/Casa de Oswaldo Cruz/Fiocruz. Rio de Janeiro – RJ – Brasil luiz.teixeira@fiocruz.br

**Keywords:** Epidemic intelligence, Public health surveillance, International Health Regulations, Inteligência epidemiológica, Secretaria de Vigilância em Saúde, Regulamento Sanitário Internacional

## Abstract

This article addresses the Brazilian government’s response to the covid-19 pandemic, particularly the public health surveillance and epidemic intelligence system. It traces the evolution of disease surveillance as a response to the International Health Regulations in the context of global health. Executive orders published in the official gazette, Diário Oficial da União, are analyzed, as well as the actors and groups formed to tackle the pandemic between January 2020 and March 2022. The founding assumption is that epidemic intelligence must be placed at the service of public health. Bureaucratic tension and changes in protagonism among different groups can be observed as these intelligence mechanisms were dismantled.

## Resumo

O artigo aborda a resposta do governo brasileiro à pandemia da covid-19, enfatizando o sistema de vigilância em saúde e de inteligência epidemiológica. Retoma a evolução da vigilância em resposta às normas do Regulamento Sanitário Internacional, no contexto da saúde global. Analisa os atos do Executivo publicados no Diário Oficial da União e se detém nos atores e grupos formados para o enfrentamento da pandemia da covid-19 de janeiro de 2020 até março de 2022. Parte da premissa de que a inteligência epidemiológica deve estar a serviço da saúde pública. Constata-se que certo tensionamento burocrático e a transferência de protagonismo entre grupos marcam o desmonte dos mecanismos de inteligência.

A eclosão da pandemia da covid-19 ampliou o debate de pautas tradicionais e trouxe novos temas para a agenda da saúde global, como o papel de cooperações para a organização de campanhas de vacinação em massa, a utilização de substâncias não reconhecidas pelas instâncias científicas, os tensionamentos entre o global, o nacional e o local na gestão de emergências sanitárias e, especialmente no caso brasileiro, tensões nas burocracias públicas e diferentes instâncias de poder. O contexto epidêmico fez com que a estrutura institucional e conceitual da saúde global, alvo de revisões críticas nas últimas décadas, passasse a ser questionada uma vez mais quanto às suas possibilidades e limitações, com apontamentos de mudanças importantes na dinâmica multilateral da saúde (Biehl, Petryna, 2013; Cueto, 2015; Cousins et al., mar. 2021).

Nesse cenário, os sistemas de vigilância em saúde receberam grande atenção no debate público, seja pela demanda por respostas coordenadas à crise sanitária provocada pelo novo coronavírus, seja pela produção de dados e modelos úteis ao planejamento dessas respostas. Para identificar, monitorar e estabelecer medidas de enfrentamento no combate às doenças emergentes, reemergentes e desconhecidas, fortemente relacionadas à circulação de pessoas, animais e objetos, é fundamental a existência de sistemas de inteligência epidemiológica bem estruturados. Segundo Waldman e Rosa (1998), inteligência epidemiológica é a aplicação plena da epidemiologia na saúde pública, considerando, nos termos de Rodrigues-Júnior (2012), uma reorganização dos serviços de saúde nas dimensões multifatoriais e geopolíticas.

No Brasil, a trajetória da vigilância em saúde remete à própria organização da saúde pública, acompanhando as preocupações com o fluxo portuário nas cidades litorâneas de maior porte, desde o início do século XX até a criação da Agência Nacional de Vigilância Sanitária, em 1999, principal ponto de inflexão nessa trajetória. Nesse percurso, o desenvolvimento do campo da saúde, com a criação de instituições e grupos profissionais dedicados à produção de dados, ao planejamento em saúde e a intervenções epidemiológicas ampliaram o sistema de vigilância em saúde no país, formado pelo Sistema Nacional de Vigilância em Saúde e pelo Sistema Nacional de Vigilância Sanitária (Brasil, 2013), ao mesmo passo em que procedimentos internacionais se consolidavam para a estandardização dos mecanismos de inteligência epidemiológica, caso dos Regulamentos Sanitários Internacionais (RSI) (Cueto, 2015).

O incremento da globalização e o consequente aumento do fluxo comercial trouxeram novos desafios para a saúde global exigindo uma revisão do RSI em 1995 (Menucci, 2006; Aith 2019). A complexidade do tema e os surtos pandêmicos de ebola, influenza, síndrome respiratória aguda grave (SARS) fez com que em 2018 fosse criado o órgão independente Global Preparedness Monitoring Board, da Organização Mundial da Saúde (OMS) com o Banco Mundial. O primeiro relatório do grupo – "O mundo em risco" – previa que uma gripe do tipo da espanhola poderia se espalhar em 36 horas, destruindo 5% da economia global (Nitahara, 22 set. 2019). O previsível retorno de uma pandemia de grandes proporções também reforçou a percepção dos Estados quanto à necessidade da existência de burocracias capazes de produzir inteligência epidemiológica, respondendo com políticas públicas adequadas às emergências sanitárias.

Nos últimos anos, principalmente desde o início do governo de Jair Bolsonaro em 2019, o sistema de vigilância em saúde no Brasil tem sofrido mudanças significativas em sua gestão e capacidade de atuação, parte de um processo mais amplo de desregulação, desde a redução do aparato estatal ao desmonte dos serviços de saúde pública. A resposta estatal à emergência sanitária da covid-19, especialmente em 2020 e 2021, tornou mais nítidos os impactos da agenda neoliberal do governo federal no funcionamento do Sistema Único de Saúde (SUS), notadamente pela pressão imposta às instituições e aos profissionais de saúde.

Ao mesmo tempo, o processo epidêmico apresentou as contradições e tensões entre os posicionamentos políticos da administração federal e as diretrizes técnicas produzidas pelas instituições de saúde, colocando em xeque a capacidade de o Estado brasileiro fomentar ações coletivas no contexto de alta vulnerabilidade sanitária. A troca de quatro ministros da saúde no período, a ocupação de cargos pelos militares, a atuação do presidente Jair Bolsonaro, que publicamente defende uma agenda contrária à das instituições de saúde pública, são alguns dos elementos indicativos que reforçam essa contraposição (Gonçalves, 2020). No âmbito político, a força dessa estratégia de cooptação e coerção interveio no funcionamento de setores em que a presença regulatória do Estado se faz necessária, como na vigilância em saúde.

Este artigo reflete sobre a formação dos grupos governamentais responsáveis pelo planejamento das ações no enfrentamento da pandemia da covid-19, a partir do acionamento do Centro de Operações de Emergência em Saúde Pública, centrando o debate em torno do sistema de vigilância em saúde e da inteligência epidemiológica. Temos como pressuposto o fato de que o enfrentamento da pandemia da covid-19 no país, no que se refere às ações de vigilância, nos seus termos mais gerais, se moldou a partir dos embates entre instituições de saúde – tradicionais ou recém-criadas – e as forças políticas e econômicas que passaram a dominar o espaço público no contexto do governo Bolsonaro. Em termos mais específicos, consideramos, nesse mesmo cenário, a atuação de diferentes burocracias técnicas que atuaram tanto como partes do sistema de saúde quanto como organismos constituídos por "burocracias profissionais" (Mintzberg, 1995), que se fizeram representar na ampla cena de construção das ações e políticas de enfrentamento da pandemia pelo SUS. A análise contempla a historicidade da formação do sistema de vigilância em saúde, refletindo acerca de mudanças e permanências em sua trajetória, principalmente em relação ao papel institucional da Secretaria de Vigilância em Saúde. Por último, realiza-se uma reflexão sobre as premissas estabelecidas a partir dos achados da pesquisa.

As fontes de pesquisa, o principal conjunto documental explorado neste artigo, referem-se aos atos publicados no *Diário Oficial da União*, no período de 1 de janeiro de 2020 a 28 de fevereiro de 2022. O processo de busca envolveu três termos: Grupo Executivo Interministerial, Comitê de Crise e Secretaria Extraordinária de Enfrentamento à Covid-19, sendo analisados publicação, data, organização principal e agentes responsáveis. A análise destacou a formação dos grupos para o enfrentamento da pandemia, os objetivos, o órgão emitente e o papel da Secretaria de Vigilância em Saúde em cada contexto. Foram analisados os atos dos poderes Executivo e Legislativo, da Presidência da República, do Ministério da Saúde e do Tribunal de Contas da União. Alguns achados foram reportados com informações veiculadas na mídia, sendo efetuada a contextualização com a literatura sobre o campo da vigilância em saúde e as emergências sanitárias.

## A vigilância em saúde no Brasil e as respostas a emergências sanitárias

Embora ações de monitoramento e vigilância remetam à formação dos aparatos de saúde pública ainda no Brasil do século XIX, a criação de um sistema de vigilância epidemiológica ocorreu somente na segunda metade do século XX, quando era recorrente o diagnóstico por parte de médicos sanitaristas de que o país necessitava de estruturas de coleta e produção de dados epidemiológicos regulares e confiáveis para o planejamento do setor da saúde (Paim, 1975). Além das limitações dos serviços de estatísticas vitais e dos recursos humanos para elaboração de estudos epidemiológicos, era apontada a resistência de médicos em realizar a notificação de doenças, mesmo aquelas listadas para notificação compulsória pelo Ministério da Saúde (Barbosa, dez. 1967). O cenário de subnotificação e de demanda por um sistema de vigilância epidemiológica tornou-se mais explícito após a ocorrência da epidemia de meningite entre 1971 e 1974, crise sanitária intensamente ocultada pelo regime ditatorial que vigia no país na época, como uma estratégia de manutenção da imagem positiva que o governo criara para si em torno do “milagre econômico” (Barata, 1988). A urgência de um sistema de vigilância no país aflorou na quinta Conferência Nacional de Saúde, em 1975, sendo debatidos o papel central do Ministério da Saúde no combate às grandes endemias. Na ocasião, foi requerido dos agentes governamentais a reorganização do Programa Nacional de Imunizações, a notificação compulsória de doenças, pilares do futuro Sistema Nacional de Vigilância Epidemiológica (SNVE), que passou a vigorar no ano seguinte (Brasil, 2017).

Os primeiros anos de atuação do SNVE foram marcados por dificuldades operacionais, em parte ligadas aos próprios desafios relacionados à implantação do Sistema Nacional de Saúde (Torres, 2020). Relacionava-se também a uma avaliação, presente no nascente movimento pela reforma sanitária, que considerava a nova política, ainda, fragmentada e sem capacidade institucional para fazer frente às demandas de um sistema de saúde moderno. Além disso, de modo geral, as estruturas de vigilância nos serviços de saúde possuíam limitações quanto aos recursos humanos e à capacidade de monitoramento e produção de dados (Castiel, 1989).

Em que pesem os consideráveis desafios políticos e institucionais, com a retomada da democracia, a partir de 1985, registramos um ambiente mais favorável às primeiras tentativas de se reduzir a influência dos interesses comerciais sobre a burocracia especializada. A chegada de Fernando Collor ao poder, em 1990, com a agenda da reforma administrativa, representou um importante retrocesso na capacidade regulatória do Estado. Segundo Eric Rosas (1994, p.62), secretário de Vigilância Sanitária, o sistema viveria o retorno do “grande e ineficiente cartório, de novo um balcão de negócios, onde a pressão de grupos políticos ou empresariais era determinante para liberação de produtos”.

A organização do SUS, a partir de 1990, introduziria mudanças conceituais e institucionais importantes no arcabouço em torno do antigo SNVE. Em termos conceituais, as atividades de vigilância passariam a ser cada vez mais compreendidas com base em um conceito amplo de saúde, assim evoluindo de uma vigilância de pessoas e doenças para uma vigilância que passava a considerar os riscos à saúde (Teixeira et al., 2018). Já em termos institucionais, a partir de 1999, com a criação da Agência Nacional de Vigilância

Sanitária, foi sendo conformado o Sistema Nacional de Vigilância Sanitária, e em 2003, com a criação da Secretaria de Vigilância em Saúde do Ministério da Saúde, o Sistema Nacional de Vigilância em Saúde. Ambos os sistemas vêm fortalecendo a descentralização das ações do campo da vigilância em saúde (Brasil, 2017).

De todo modo, para alguns autores, em que pesem os avanços conceituais e institucionais, a fragmentação existente nos sistemas de vigilância em saúde continuaria a representar um problema para um adequado planejamento das políticas de saúde, e se constitui, ainda hoje, como parte de um conjunto de desafios a ser enfrentado pela gestão do SUS (Aith, Dallari, 2009; Aith, 2019).

Nesse mesmo contexto, o papel de instrumentos padronizados de vigilância se consolidava no âmbito da saúde internacional, impulsionado pelo ideário da saúde global emergente na década de 1990 e pela ampliação da relevância de métricas epidemiológicas no planejamento de políticas públicas e nas tomadas de decisão em saúde (Adams, 2016). Dessa forma, ainda que sob imensos desafios, a atuação do sistema nacional de vigilância brasileiro aproximava-se cada vez mais de parâmetros internacionais guiados pelo processo de estandardização no campo da saúde. Em 2005, as recomendações do RSI foram aprovadas na 58ª Assembleia Mundial da Saúde, tornando-se a principal referência dos sistemas de saúde para o monitoramento e controle de epidemias e pandemias (Menucci, 2006). A OMS de forma estratégica fomenta a criação de Pontos Focais Nacionais que passam a atuar como referência dos países no sistema de monitoramento e vigilância da saúde global (Haustein et al., 2011; Packer et al., 2021). É importante enfatizar, nesse caso, a relevância da autonomia e a capacidade técnico-gerencial necessária para atuação desses representantes nacionais que operam em articulação global, desenvolvendo a capacidade do Estado em estabelecer um efetivo sistema de inteligência epidemiológica.

O destaque conferido ao conceito da Emergência em Saúde Pública de Importância Internacional, por parte do novo ordenamento internacional, demandava a existência de uma resposta nacional integrada com vistas ao atendimento das demandas da saúde global (Menucci, 2006). Com esse intuito, desde 2006, foi sendo instituído um sistema de tecnologia própria em todo o território brasileiro, conhecido como a Rede Nacional de Alerta e Resposta às Emergências em Saúde Pública – Rede Cievs (Carmo, Penna, Oliveira, 2008; Brasil, 2016).

Em maio de 2009, após a OMS emitir um alerta de ESPII devido ao surto epidêmico do vírus da influenza A (H1N1), conhecido popularmente como gripe suína, a Secretaria de Vigilância em Saúde elaborou o plano de preparação para o enfrentamento da pandemia no país (Matos, 2011). Ainda em julho, o Congresso Nacional reconheceu as normas do RSI, por meio do decreto legislativo n.395 (Senado Federal, 13 mar. 2009).

Pode-se considerar que, em situações de risco sanitário iminente, a conformação de um grupo de atores, a exemplo do Grupo Executivo Interministerial, tornava-se estratégica para a tomada de decisões e desenvolvimento de ações articuladas. Nesse sentido, a experiência do enfrentamento à epidemia causada pelo vírus H1N1 marcou a primeira organização desse grupo, sob coordenação da Secretaria de Vigilância em Saúde. Gerson Penna, então secretário da pasta, e José Gomes Temporão, ministro da Saúde, reforçaram na época o papel institucional que o grupo teria na aplicação das ações do Plano de Emergência, estabelecido em alinhamento com a agenda internacional em curso, por meio da Organização Pan-americana da Saúde (Couto, 5 maio 2009).

Um ano após o enfrentamento da gripe suína, em 2010, foi instituído o Grupo Executivo Interministerial de Emergência em Saúde Pública de Importância Nacional e Internacional (GEI-ESPII), estando a cargo da Secretaria de Vigilância em Saúde a coordenação das ações dessa instância política (Brasil, 2010). O GEI-ESPII seria acionado, novamente, em 2015, diante do surto de microcefalia decorrente do vírus da zika (Brasil, 2015). Essa emergência sanitária nacional foi reconhecida mundialmente e levou a um conjunto de ações articuladas internacionalmente. Nesse contexto, a Secretaria de Vigilância em Saúde produzia boletins epidemiológicos periódicos, respondia pelas ações e os "próximos passos" do Plano Nacional de Enfrentamento à Microcefalia. Segundo Leila Garcia (2015, p.33):

> A rápida resposta à emergência em saúde pública – com a investigação dos casos e fatores associados, a notificação às autoridades sanitárias internacionais e o desencadeamento das ações de vigilância, prevenção e assistência à saúde de forma articulada –, em menos de dois meses após a detecção do surto de microcefalia, foi possível somente devido à operação de um sistema de vigilância eficiente, no âmbito do Sistema Único de Saúde (SUS) no Brasil.

A resposta brasileira no enfrentamento da microcefalia deu origem ao Centro de Operações de Emergência em Saúde Pública (COE). A burocracia em torno do COE deve atuar em toda emergência sanitária nacional, incluídos os desastres naturais. Nessa linha de ação, a Força Nacional do SUS foi criada para atuar em articulação com a Defesa Civil (Silva et al., 2020). A esse respeito, convém sinalizar que todo o aparato burocrático responsável pelo planejamento dessas ações passou a exigir do Estado brasileiro, de forma crescente, maior capacidade de coordenação, integração, transparência e parceria com a sociedade. Sob esses marcos, nos primeiros meses de 2020, um evento sanitário global dramático não só testaria a conformação do sistema de vigilância em suas recentes transformações históricas, mas introduziria importantes mudanças e tensões em sua gestão.

## O governo Bolsonaro e a resposta brasileira à pandemia da covid-19

Desde 2013, o cenário político-institucional brasileiro tem experimentado um recuo considerável na agenda progressista estabelecida na virada do século XX para o XXI e um fortalecimento de pautas associadas à extrema-direita populista e a projetos de desmonte do Estado a partir da perspectiva neoliberal (Santos, Feldmann, 2021). Após um golpe realizado mediante o *impeachment* da presidente Dilma Rousseff, em 2016, o programa neoliberal se tornou pauta principal nos governos de Michel Temer e de Jair Bolsonaro, o último iniciado em 2019. Somado a isso, a administração de Bolsonaro trouxe uma enorme presença de militares – da ativa e da reserva – para a gestão do Estado, contorno bastante característico de um governo populista de extrema-direita com inclinações autoritárias.

Ainda em 2019, diversas tensões produzidas pelo presidente da República criaram um cenário de instabilidade institucional cujos impactos foram visíveis na condução da resposta à pandemia da covid-19. Três aspectos gerais do ambiente político podem ser destacados de partida: a constante suspeição e desafio à autoridade institucional do Poder Judiciário, em especial o Supremo Tribunal Federal; os questionamentos e parcial ruptura do pacto federativo, principalmente quanto a estados governados por políticos de oposição; e o estabelecimento de uma diretriz de diminuição da máquina pública, sobretudo em pontos centrais das políticas sociais, a exemplo da extinção do Ministério do Trabalho (restituído em 2021). No esteio da ameaça sanitária anunciada pelo novo coronavírus, no início de 2020, o cenário político brasileiro apresentava grande instabilidade e pouco espaço para ações articuladas entre os três poderes da administração federal e entre o governo central e as outras esferas da federação.

A Síndrome Respiratória Aguda Grave causada pelo novo coronavírus teve início na cidade de Wuhan, na China – epicentro inicial – levando o governo chinês a decretar quarentena em consequência do índice de propagação e do número de casos. Em fins de janeiro de 2020, a OMS emitia uma declaração de emergência sanitária e, segundo às normas do RSI, os sistemas de saúde deveriam estabelecer as medidas de enfrentamento para a nova doença (OMS, 2020a). As operações logísticas e comerciais de portos e aeroportos de fronteiras ainda não estavam de todo no radar das medidas de segurança. Segundo o diretor geral da OMS, Tedros Adhanom, era importante que os Estados passassem a seguir as recomendações do Comitê de Emergência do RSI para impedir a propagação do vírus e garantir uma resposta adequada, baseada nas melhores evidências científicas (OMS, 2020b).

No Brasil, nesse mesmo contexto, o Ministério da Saúde preocupava-se com o avanço dos registros da nova doença e com o aumento dos casos de sarampo (Mandetta, 2020). Em 22 de janeiro, a equipe do Centro de Operações de Emergência foi acionada para o enfrentamento do novo coronavírus (2019-nCoV), sendo designada como COE-nCoV (Croda, Garcia, 2020).

Convém observar, entretanto, o cenário de incertezas que permeava as primeiras respostas sanitárias à nova doença causada pelo SARS-CoV-2. O que se sabia acerca do mecanismo de transmissão, observando-se a importância da exposição das vias aéreas, mobilizava enunciados já estabelecidos pela medicina e pela saúde pública. O contato de pessoa a pessoa convocava cuidados quanto às situações de confinamento, por exemplo. Por outro lado, de início observamos incertezas quanto ao uso em massa de máscaras de proteção, cujas orientações sofreram variações consideráveis em intervalos de meses. Nesse sentido, a tomada de decisões baseada em evidências partia de um terreno relativamente movediço, que em parte considerava enunciados já estabelecidos acerca dos mecanismos de contágios de doenças respiratórias e em parte se situava numa profusão de novos estudos que realizavam um gerenciamento das dúvidas relativas à covid-19, nome dado à nova doença.

Nesse contexto inicial, estavam entre as principais recomendações da OMS (2020b): contenção da epidemia por meio do acionamento do sistema de vigilância em saúde; detecção precoce; isolamento e gerenciamento de casos; rastreamento de contatos; e prevenção da disseminação progressiva da infecção. O cenário complexo exigia o compartilhamento de dados com a OMS e fomentava as trocas institucionais em relação às melhores evidências científicas no enfrentamento da nova doença. No mesmo dia da declaração de Emergência Sanitária pela OMS, o governo criava o Grupo Executivo Interministerial (Brasil, 2020a).

O acionamento do Centro de Operações de Emergência e a formação do Grupo Executivo sinalizavam, inicialmente, que o governo seguia com as mesmas práticas institucionais que haviam levado o país para o grau de resposta no enfrentamento da zika em 2016.

O Ministério da Saúde publicava as primeiras medidas de enfrentamento da Emergência de Saúde Pública de Importância Nacional no início de fevereiro (Brasil, 2020b). A lei n.13.979, instituída três dias após a portaria, indicava para certa convergência de ações entre governo e Congresso Nacional, reforçando as práticas recomendadas para todas as organizações (Brasil, 2020c). Outra ação do Poder Executivo, no mesmo dia, chamou a atenção: o presidente e o ministro da Economia publicaram a medida provisória n.921 (Brasil, 2020d), solicitando o crédito extraordinário para o Ministério da Defesa no valor de R$ 11.287.803,00, destinando recursos para as medidas de enfrentamento ao novo coronavírus. Esse dado aponta para certo protagonismo da agenda do Ministério da Defesa em detrimento do planejamento de ações sugeridas pela Saúde.

Na prática observamos uma crescente sobreposição de atribuições e disputas de interesses no interior da burocracia estatal. A incorporação das Forças Armadas de maneira incisiva em pautas relacionadas à esfera civil tem sido um traço característico do governo, algo representado pela já mencionada presença numerosa de militares em cargos da administração federal, desde os ministérios até as empresas estatais. No caso da resposta à covid-19, a atuação do Ministério da Defesa trazia uma perspectiva distinta daquela mobilizada pela burocracia da Saúde, pois a Defesa tinha como base os investimentos públicos (financeiros e políticos), despendidos pelo chefe do Executivo, em favor da massificação do uso da hidroxicloroquina no tratamento da doença. Tal orientação política se distanciava das diretrizes técnicas, embasadas em evidências científicas elaboradas por diversas sociedades médicas – como a Sociedade Brasileira de Infectologia, a Sociedade Brasileira de Pneumologia e Tisiologia e a Sociedade Brasileira de Imunologia – e, também, contrariava as instâncias de participação social do SUS, visto que o Conselho Nacional de Saúde se posicionou de forma contrária ao uso da hidroxicloroquina como terapêutica para a covid-19. Para alguns autores, essa forma de lidar com a gestão de crises sanitárias caracterizaria um retorno ao *modus operandi* que os militares utilizaram na gestão da saúde no período da ditadura (Souza, 2020).

A partir desse momento, por volta de março de 2020, o tensionamento entre a agenda da saúde global e o contexto nacional, bem como, no plano interno, entre o Executivo Federal e os órgãos técnicos da Saúde, tornou-se mais explícito, principalmente com a negação do próprio presidente quanto à gravidade da nova doença. Em pronunciamento à população no dia 24 de março, duas semanas após a declaração da pandemia de covid-19 pela OMS, Bolsonaro negou veementemente o impacto da doença e iniciou uma estratégia discursiva central à resposta do governo federal, elaborando uma falsa simetria entre saúde e economia. Acusando os meios de comunicação de promover histeria na população, Bolsonaro afirmava que "noventa por cento de nós não teremos qualquer manifestação caso se contamine" e, em uma passagem marcante, comentou que no seu caso, devido ao "histórico de atleta", o vírus causaria uma "gripezinha" (Brito, 24 mar. 2020). A fala do presidente, naquele momento, já colocava uma contraposição central no processo epidêmico vivenciado no Brasil: entre medidas preventivas não medicamentosas, sobretudo o isolamento social/*lockdown*, e o uso de medicamentos apontados como soluções imediatas para a doença.

Dessa forma, o discurso de Bolsonaro traduzia os limites governamentais do país no enfrentamento de uma emergência sanitária internacional. Mais que isso, colocava em risco os processos de articulação institucional instituídos, a duras penas, tanto em nível nacional como internacional. Os objetivos do Grupo Executivo formado em 2020, sem a condução do governo federal, eram, na prática, cada vez mais limitados (Brasil, 2010, 2020a). A desarticulação com os movimentos sociais e o permanente ataque às demais instituições democráticas, por parte do governo, produziram visíveis consequências para a desorganização institucional da resposta brasileira à pandemia (Shimizu et al., 2021). Os tensionamentos institucionais ficariam mais evidentes a partir de mais um aparato institucional, desta vez, sob o comando dos militares: o Comitê de Crise para Supervisão e Monitoramento dos Impactos da Covid-19. Tornava-se, assim, evidente a transferência do protagonismo da Secretaria de Vigilância em Saúde à frente do Centro de Operações de Emergência, para o ministro-chefe da Casa Civil (Brasil, 2020e).

Não tardaria para o ápice da tensão no interior da burocracia ser expresso pela demissão de Luiz Mandetta, ministro da Saúde no período. Segundo Schreiber (17 abr. 2020), "O presidente Jair Bolsonaro fez valer o peso de sua caneta, como havia ameaçado, ... após um mês de embates sobre a melhor estratégia para o enfrentamento da pandemia de coronavírus".

Apesar da divisão institucional no interior do governo ser questionada pelo plenário do Tribunal de Contas da União (TCU, 7 jul. 2020), a responsabilidade na condução da pandemia recaiu sobre os entes federados, impactando diretamente a gestão dos serviços de saúde. Desse modo, em razão da insegurança política-institucional provocada deliberadamente pelo governo, o país adoecia diante do número crescente de novos casos de covid-19.

## O sistema de vigilância sob o comando dos militares

O agravamento dos impactos da pandemia de covid-19 no Brasil acompanhou os movimentos de realinhamento político da administração de Jair Bolsonaro, sobretudo no que tange ao papel exercido pelas Forças Armadas (principalmente o Exército) nas operações do governo e na articulação com outros poderes da República. Duas figuras foram relevantes nesse processo, os generais da ativa Eduardo Pazuello e Walter Braga Netto. O primeiro ocupou o posto de ministro da Saúde entre maio e setembro de 2020 de forma interina e, desse momento até março de 2021, na condição de titular da pasta; o segundo chefiou a Casa Civil da Presidência entre fevereiro de 2020 e março de 2021, quando foi alçado ao cargo de ministro da Defesa. É importante observar que, na organização da carreira militar, oficiais que se encontram na ativa possuem vínculos mais estreitos e explícitos com as diretrizes e ideologias do Exército, o que, inclusive, impõe limitações legais à sua atuação na esfera política. Dessa forma, a incorporação de oficiais de alta patente em postos centrais da administração federal representou um alinhamento importante entre as agendas do presidente e do alto comando do Exército, um tipo de movimentação política que remete, mais uma vez, ao período da ditadura militar.

Alguns dias após a criação do Comitê de Crise, foi designado o Centro de Coordenação de Operações, atuando como um subcomando dentro do Comitê. Entre algumas de suas ações, o subcomando deveria coordenar e monitorar as operações de enfrentamento da covid-19 com os entes públicos e privados e as medidas estabelecidas pelos governadores, mantendo atualizado o núcleo central do governo sobre o avanço da pandemia. Toda a conformação desse "subgrupo" foi designada pelo general Braga Netto, estando à frente Heitor Freire de Abreu, um dos assessores do ministro da Defesa (Brasil, 2020f). Uma das ações publicizadas do subcomando foi a realização, em junho, do primeiro Workshop de Boas Práticas no Enfrentamento da Covid-19 (Casa Civil, 2 jun. 2020).

A ampliação da presença de militares no MS foi considerada "o escândalo da desinstituição da autoridade sanitária do SUS" por atores e instituições do campo da saúde (Santos, Guimarães, 2020, p.74). Além disso, é um caso significativo do entrelaçamento de interesses e perspectivas na gestão do setor da saúde, uma demonstração superlativa de como a organização do setor não estava disposta estritamente pelo caráter técnico, uma "saúde pública baseada em evidências" (Adams, 2013) como se defende desde o início da implantação do SUS. No caso da resposta brasileira à pandemia, a relação entre técnicos do MS e grupos associados à Presidência da República chegou próxima à cisão em alguns momentos. Um elemento significativo desse processo foi o estabelecimento de canais de comunicação paralelos dos círculos mais próximos do presidente em relação à comunicação oficial do ministério e de suas instituições. Esse processo gerou um quadro emblemático de embaraço institucional, em que alguns órgãos do governo federal, como a Fundação Oswaldo Cruz, forneciam à população informações que eram criticadas e combatidas pelos canais mais circunscritos ao presidente, como a Secretaria de Comunicação da Presidência.

Para a sociedade, a ausência de um canal público e transparente sobre a pandemia e a divulgação de dados confiáveis da vigilância epidemiológica estabelecida em décadas de SUS é um apagamento silencioso e desafiador para a gestão do sistema de saúde. A mídia tratou de formar um consórcio com base nos registros das Secretarias de Saúde dos estados federados. A produção de informação tornou-se objeto de disputa dos veículos de comunicação e redes sociais, contribuindo para a confusão e insegurança da população. Como abordado, a inteligência epidemiológica deve servir ao direcionamento das políticas públicas, o "apagão estatístico" que marca a atuação do governo na pandemia abalava os pilares do sistema de vigilância em construção (Oliveira, 2020).

Observou-se, ainda, um processo generalizado de formação de Comitês de Crise, entre as mais diferentes instituições públicas e privadas, sendo compostos de diversos perfis de técnicos e de políticos (Shimizu et al., 2021). De todo modo, como parte da resposta da sociedade civil organizada e dos principais canais de comunicação, os epidemiologistas e demais profissionais da saúde foram se tornando referências públicas que teciam análises sobre a evolução da covid-19 e sobre as medidas de enfrentamento. Algumas dessas pessoas, tais como a médica infectologista Margareth Dalcomo, da Fundação Oswaldo Cruz, se tornariam figuras nacionalmente reconhecidas e publicamente laureadas.

Ainda em junho, Braga Netto nomearia Arnaldo Correia de Medeiros para a pasta da Secretaria de Vigilância em Saúde (Brasil, 2020g). Arnaldo foi indicado pelo Centrão – bloco informal de parlamentares conservadores que historicamente buscaram estar próximos ao

Poder Executivo para garantir vantagens fisiológicas. Segundo a mídia, o nome indicado se deve à crise política instalada pela condução do governo no enfrentamento da pandemia (Cancian, Fernandes, 5 jun. 2020). O codinome COE-nCoV que produzia os boletins epidemiológicos seria extinto a partir da entrada de Arnaldo (Mota, Teixeira, 2020).

O subcomando do Comitê de Crise torna-se, de fato, a referência central do governo conforme constatado nas atas do Tribunal de Contas da União (TCU). No dia 24 de junho, o TCU questiona a inexistência de um sistema de governança em relação à pandemia. No período, a média de mortes estava "oficialmente" em mil brasileiros por dia. Os ministros do TCU alertavam à Casa Civil da Presidência da República:

> a ausência de diretriz estratégica clara de enfrentamento à covid-19, com a respectiva gestão de riscos, bem como a ausência de um plano de comunicação coordenado e abrangente, pode comprometer os gastos e os resultados do enfrentamento à pandemia e impedir uma efetiva coordenação política e articulação entre órgãos e entidades, federais e subnacionais (TCU, 7 jul. 2020).

Além da constatação, o TCU recomendou que fossem incluídos outros atores relevantes no Comitê, a exemplo dos: "presidentes do Conselho Federal de Medicina, da Associação Médica Brasileira e do Conselho Nacional de Secretários de Saúde", dando direito a voz e a voto, além da Secretaria Especial de Comunicação Social do Ministério das Comunicações. O TCU (7 jul. 2020) também recomendou que fossem publicizadas as atas das reuniões deste Comitê, conforme exige o Princípio da Transparência.

Em outubro, a Casa Civil ratificou as diretrizes do Comitê de Crise no papel central do governo na pandemia (Brasil, 2020h). Observa-se no documento que o governo reconhece a necessidade de uma articulação com representantes da gestão do SUS, citando o Conselho Nacional de Secretarias Municipais de Saúde e o Conselho Nacional de Secretários de Saúde. A articulação deveria ser efetuada por meio do MS, "quando possível e necessária", para "manter uma relação de confiança e acompanhamento das necessidades". Tais diretrizes representavam, sem dúvida, uma inversão de valores da gestão do SUS, uma vez que sugeriam um papel subalterno, senão alternativo, entre as diversas instâncias de um sistema descentralizado de saúde no processo de planejamento e formulação de políticas públicas.

Em dezembro de 2020, mais um grave capítulo na trajetória da pandemia da covid-19 no Brasil. Nesse mês, na cidade de Manaus, a falta de suprimento de cilindros de oxigênio levou várias pessoas à morte por asfixia relacionada à covid-19 e escancarou ainda mais os problemas da resposta governamental. O caso dramático da capital do Amazonas gerou grande mobilização, repercutindo, inclusive, no estabelecimento de uma Comissão Parlamentar de Inquérito (CPI) no Senado Federal, cujo objetivo foi investigar a gestão do governo federal durante a pandemia.

Entre a crise de Manaus e o início da CPI, em abril de 2021, uma troca de comando no Ministério da Saúde sugeria outros alinhamentos políticos no governo federal. O cardiologista Marcello Queiroga, ex-presidente da Sociedade Brasileira de Hemodinâmica e Cardiologia Intervencionista, assumiu o comando da pasta, enquanto Braga Netto passou da Casa Civil para a Defesa. Essas trocas foram uma reação do governo a pressões políticas e sociais acerca de uma gestão de crise sanitária que foi escrutinada nos meses seguintes pela CPI, que atestou que, sob o comando de Braga Netto no Comitê de Crise:

> As ações e inações do ministro Braga Netto e do ex-coordenador Heitor Freire de Abreu são, portanto, suficientes para configurar a possível prática do crime de epidemia, considerando seu dever de agir e a relevância das suas omissões ao quedarem-se inertes e, assim, contribuírem para o agravamento da pandemia (Senado Federal, 2021, p.260).

Mesmo com a saída de Braga Netto da Casa Civil, o Comitê de Crise se manteve ativo, como constatado nas atas do TCU, ainda que tenha sido observada uma redução de atos publicados em comparação com os meses anteriores. Queiroga, assumindo o comando da gestão da pandemia, alteraria a estrutura do Ministério da Saúde criando a Secretaria Extraordinária de Enfrentamento à Covid-19 (Secovid), ligada diretamente ao seu gabinete (Brasil, 2021a). A Secovid assume o planejamento de ações no enfrentamento da pandemia da covid-19, em paralelo à instalação da CPI da Covid-19. Na tentativa de minimizar a crise instalada na saúde, buscava-se dar transparência às ações do governo e estabelecer uma importante articulação com representantes de diferentes organizações da saúde tanto do setor público como do privado. O governo direcionava seus esforços para minimizar um conjunto de denúncias sobre a gestão de Pazuello no Ministério da Saúde, divulgadas no relatório da CPI da Covid-19, e na organização da vacinação contra a covid-19.

## A campanha de vacinação e a Secretaria Extraordinária de Enfrentamento à Covid-19

Em 2021, a autorização emergencial para uso das vacinas produzidas pelo Instituto Butantan (Coronavac) e pela Fundação Oswaldo Cruz (Astrazeneca) deu início ao processo de vacinação da população brasileira. Com a introdução das vacinas na resposta ao processo epidêmico, o debate público foi reformulado – com a ampliação da pressão para compra de imunizantes e articulação para a adesão da população – e a atenção se voltou cada vez mais para as métricas da imunização (Camargo et al., 2021). Por sua vez, a intensificação dos “nacionalismos vacinais” (Fidler, 2020), expressão referente ao movimento de países mais ricos para garantir a compra de imunizantes para suas populações em detrimento dos países mais pobres, tornou mais nítida a fragilidade de ações coordenadas de vigilância e prevenção em termos da saúde global.

No Brasil, a especificidade localizou-se no enfrentamento entre a pressão pela aquisição, distribuição e aplicação de vacinas e a insistência do governo federal – respaldado pelo Conselho Federal de Medicina – em propagar o uso de medicamentos para o tratamento da doença, a despeito da falta de reconhecimento da eficácia das drogas pela comunidade médico-científica. Embora a gestão de Marcello Queiroga tenha aderido formalmente ao imperativo da vacinação, a persistência do “kit covid” como diretriz para o combate à pandemia era mantida em outros circuitos vinculados à Presidência da República, a exemplo do “Gabinete Paralelo”, que apoiava as ações governamentais, como o uso da cloroquina, evidenciado pela CPI da Covid-19 (Dias, Lima, Lobo, 2021).

Segundo noticiado pela imprensa, a médica infectologista Luana Araújo, indicada para assumir a chefia da Secovid, tinha posição contrária ao uso de hidroxicloroquina e outros medicamentos de uso *off-label* contra a covid-19. Após consultas, seu nome seria substituído pelo de Rosana Leite de Melo. Nas palavras de Marcello Queiroga, a doutora Luana "não traria a conciliação entre os médicos", ao contrário da indicação da doutora Rosana, que teve sua "validação política" pela classe médica (Ribeiro, 18 jun. 2021). Mais um caso que retratava a subordinação dos assuntos técnicos aos interesses impostos pela ideologia política na gestão da crise sanitária. No entanto, nesse momento, a escolha acabou funcionando contrariamente às diretrizes do Executivo. Embora Rosana fosse colaboradora do ministro no tempo em que atuou como secretária da Comissão Nacional de Residência Médica e aceitasse uma posição menos dura em relação à proibição da indicação da cloroquina na relação dos médicos com seus pacientes, a médica, que já havia se indisponibilizado com o ministro da Educação por suas posições políticas, mostrou-se muito mais aberta às posições dos técnicos da saúde (Costa, 17 jun. 2021).

A criticidade que marca o tempo histórico do SUS exigiu da nova secretaria uma articulação nacional em torno dos agentes do sistema de vigilância em saúde, tanto no planejamento da imunização da população quanto no monitoramento e avaliação contínua do processo da vacinação contra a covid-19. Cerca de três meses depois de sua criação, em agosto, a Secovid introduziu algumas mudanças na gestão da crise sanitária. De um lado, alterou-se a conformação da Câmara Técnica de Assessoramento em Imunização, criada em 2003. De outro, criou-se a Câmara Técnica de Imunização da Covid-19. Esta última contou com diversas entidades médicas, a exemplo do Conselho Federal de Medicina e Enfermagem, consultores *ad hoc*, técnicos de sistema de informação, entre outros atores estratégicos (Brasil, 2021b).

Ao abranger novos representantes, principalmente do setor médico, a Câmara Técnica se contrapunha à diretriz original e predominante no governo ao longo da emergência sanitária, pautada no negacionismo científico e na articulação política com os militares. Nesse sentido, prevendo o público-alvo infantil para a vacinação, a Secovid, em parceria com as entidades médicas, passou a ter papel importante no enfrentamento do negacionismo de atores contrários à vacinação contra a covid-19, especialmente nessa faixa etária. Colocava-se, assim, como contraponto, por exemplo, à deputada federal Bia Kicis (PL), uma das principais apoiadoras do governo; ou mesmo à ministra dos Direitos Humanos Damares Alves, que emitiu notas técnicas contra a obrigatoriedade da vacinação infantil (Cristaldo, 28 mar. 2022).

Dessa forma, a histórica fragmentação dos sistemas de vigilância, enfrentada sob duras dificuldades no contexto de implantação do SUS, foi agravada na condução de um governo que fracassava em fornecer bens coletivos e respeitar os cidadãos no direito à vida. Em sentido contrário, a autonomia de ação de frações da burocracia da saúde permitiu que a sanha desorganizadora do governo federal fosse em alguma medida freada no âmbito da atuação da Secovid.

## Considerações finais

Neste artigo, abordamos a resposta do governo brasileiro à pandemia da covid-19, com ênfase no gerenciamento do sistema de vigilância em saúde e de inteligência epidemiológica no país. Em um cenário permeado por condicionantes estruturais e conjunturais, a exemplo do problema da subnotificação de doenças e da emergência de governos de extrema-direita, a vigilância em saúde foi tensionada tanto no âmbito da saúde global quanto na governança dos Estados. Um pouco antes da pandemia da covid-19, pesquisadores já haviam identificado deficiências estruturais nos pontos focais dos sistemas de vigilância epidemiológica dos países: limitada integração intersetorial, acesso restrito a lideranças do governo, sistemas de informação incipientes, e a ausência de autonomia para fazer as notificações à OMS (Packer et al., 2021). Constantes reconfigurações do aparato estatal apontam para sérios riscos na condução de práticas institucionais consolidadas, a exemplo da atuação da Secretaria de Vigilância em Saúde.

Os impactos do gerenciamento tensionado, expressão do conflito entre a burocracia técnica e as instâncias políticas do governo, foram sentidos diretamente pela população, a exemplo das diversas desordens de orientação sanitária, problemas no processo de monitoramento e avaliação de dados e da retirada de autonomia de técnicos do setor da saúde. Em um sistema de vigilância cuja atuação possui limitações historicamente estabelecidas, o contraponto entre a diretriz governamental, as instituições de saúde pública e a agenda da saúde global traçou um cenário de maior fragilidade nas ações de monitoramento e informação epidemiológica. Vazamentos de informação de dados dos brasileiros, instabilidades nos sistemas de informação e a constante desatualização dos sistemas de registros de dados da pandemia revelam um alto nível de incerteza, instabilidade e conflito institucionais.

A rápida disseminação do vírus da covid-19 e suas variantes, o desalinhamento na adoção de medidas de enfrentamento de forma global e nacional, a limitada capacidade de autonomia dos estados nacionais e instâncias subnacionais e a constante interrupção na publicização dos indicadores epidemiológicos são algumas evidências que apontam como as normas, apesar de estabelecidas, não garantem por si só que os acordos possam ser cumpridos pelos governos, aumentando assim o risco para uma má gestão de eventual crise sanitária. Alguns autores apontam que a pandemia da covid-19 acabou por demonstrar limites do RSI, em situações de aumento das iniquidades sociais, incipiente regulação nos sistemas de saúde e da justiça, colocando à prova a capacidade dos Estados democráticos de direito (Gostin, Habibi, Meier, 2020), como discutido ao longo do trabalho em relação ao Brasil.

Em um quadro mais amplo, o gerenciamento tensionado da vigilância em saúde durante a pandemia de covid-19 nos coloca também questões importantes acerca dos limites da interação da saúde global com as especificidades nacionais/locais. Se o nacionalismo vacinal, tão retratado pela mídia, aponta para uma supressão de preocupações multilaterais em face dos interesses nacionais, principalmente de países mais ricos, o caso brasileiro aponta para ingredientes adicionais. Aqui o monitoramento epidemiológico e a própria oferta de imunizantes tornaram-se objeto de disputas políticas não só entre o Executivo Federal e os entes subnacionais, mas também entre o Executivo Federal e as instâncias técnicas federais e subnacionais.

O caso brasileiro, portanto, revelou que orientação de uma "saúde pública baseada em evidências", conduzida por órgãos técnicos, precisa levar em conta a dificuldade de aplicação dessas evidências em contextos sanitários específicos e também os graus de adesão, interesse e, especialmente, de conflito para essas aplicações. Como aponta Thevenot

(2009), a governança sanitária por meio de padrões (*standards*) é orientada por interesses e perspectivas situadas e situacionais, fazendo com que a adesão a respostas padronizadas e a protocolos varie bastante de acordo com o ambiente político-institucional e com as interações entre grupos e indivíduos.

Para além dos questionamentos e ponderações teórico-analíticas, procuramos mostrar que existe um quadro concreto de deterioração e desmonte dos mecanismos de inteligência em saúde, que afetam concretamente a população brasileira. A tradução mais drástica desse processo é o número de mortes por covid-19 no Brasil, que ultrapassa 668 mil pessoas que foram a óbito não somente devido à letalidade do vírus, mas, sobretudo, por problemas graves na condução da resposta pública ao processo epidêmico.

Tudo leva a crer que o desarranjo institucional para o enfrentamento de crises sanitárias, em nossos dias vigente, pode ser duradouro, transcendendo, como legado, para outros tempos e governos. Representará, no entanto, mais uma agenda de enfrentamento urgente, por parte de novos governantes, no sentido de se estabelecer tanto um Estado mais eficiente quanto uma sociedade mais justa e sintonizada com as necessidades dos cidadãos.
